# The Role of Cardiovascular Magnetic Resonance Imaging in Athletic Individuals—A Narrative Review

**DOI:** 10.3390/jcm14103576

**Published:** 2025-05-20

**Authors:** Neil Grech, Mark Abela

**Affiliations:** 1 Department of Cardiology, Mater Dei Hospital, MSD 2090 Msida, Malta; neil.b.grech@gov.mt; 2 Department of Medicine, University of Malta, MSD 2090 Msida, Malta; 3 Cardiovascular and Genomics Research Institute, City St George’s, University of London, Cranmer Terrace, London SW17 0RE, UK

**Keywords:** cardiac MRI, cardiomyopathy, sudden cardiac death, fibrosis, athlete

## Abstract

Cardiovascular magnetic resonance imaging (MRI) is an advanced cardiac imaging modality that is often required when evaluating athletic individuals. Unrestricted imaging planes, excellent spatial resolution, and a lack of ionising radiation are some of the benefits of this modality. Cardiac MRI has been established as the gold standard imaging modality for morphological assessment, volumetric analysis, and tissue characterisation. Cardiac MRI without any doubt is an excellent diagnostic tool when evaluating athletes with symptoms or those individuals exhibiting equivocal findings at screening. It is also useful for athletes who fall within the grey zone and is especially important among athletes with a suspected or confirmed diagnosis. Cardiac MRI plays a strategic role when adopting a shared decision-making model in athletes with heart disease, tailoring and personalising medical care to the condition and the athlete’s wishes. The aim of this review is to provide a comprehensive yet practical overview of the role of cardiac MRI when evaluating athletes in clinic.

## 1. Introduction

Participation in regular intense exercise undoubtedly promotes a favourable cardiovascular risk profile. Regularly engaging in physical activity also leads to a substantial benefit for most non-cardiovascular systems. Physically active individuals have a lower risk of malignancy, later onset of dementia, better glycaemic control, lower risk of fractures, and better mental health [1]. Modest amounts of exercise also confer a significant benefit. Every metabolic equivalent of tasks achieved >4 h translates into a 12–20% reduction in cardiovascular mortality [2]. Current guidelines recommend a minimum of weekly training of a volume of 150 min of moderate intensity exercise for all adults [3]. Competitive and very keen recreational athletes, however often exceed these recommendations. Fit athletes often require a sustained ×5–6-fold cardiac output to cope with the physical demands during exercise regimes [1].

The search for sporting excellence, however, comes at a cost. Sudden cardiac death (SCD) in athletes is unfortunately a reality, which is why most bodies across the Atlantic endorse cardiac screening in athletes [4,5]. Genetic or congenital disorders like cardiomyopathies, coronary artery anomalies, and ion-channel disorders are the leading causes of SCD in young athletes [6]. Coronary artery disease is, on the other hand, the predominant disease entity affecting older athletes. Most athletes harbouring these conditions are asymptomatic [7], which is why clinical suspicion of these conditions is often prompted following screening. Athletes may invariably present electively to clinic with symptoms or a family history that may be suggestive of an inherited familial disorder. Acute presentations with new symptoms, arrhythmias, heart failure, or cardiac arrest is also infrequently the case.

A comprehensive evaluation is often required when athletes are referred for assessment [8,9]. Cardiac imaging helps confirm or rule out pathology and also helps ascertain an individual’s risk. The profound athletic remodelling often seen in athletic individuals may, however, pose some challenges, hence why a whole array of tests is often required [10]. Certainly, echocardiography is an excellent imaging modality that offers an excellent spatial and temporal resolution. It is also readily available and inexpensive. Echocardiography provides both functional and structural assessments of all four cardiac chambers, valves, and the aorta. Despite these strengths, advanced cardiac imaging is now taking centre stage. Cardiovascular magnetic resonance imaging (MRI) plays a strategic role in the evaluation of athletes and is increasingly being used in day-to-day clinical practice [11]. Its excellent spatial and temporal resolution and unrestricted imaging planes have allowed it to establish itself as the gold standard imaging modality for morphological assessment, volumetric analysis, and tissue characterisation [12]. The role of this manuscript is to provide a comprehensive overview of the role of cardiac MRI when evaluating athletes in day-to-day clinical practice.

## 2. Clinical Indications for Cardiac MRI in Athletes

Cardiac MRI, without any reasonable doubt, provides an excellent morpho-functional assessment of the heart. Tissue characterisation with cardiac MRI has revolutionised our understanding of the athlete’s heart. This imaging modality has also provided excellent insight into various pathological entities that may present in athletes. It also plays a strategic role when adopting a shared decision-making approach in athletes with a diagnosis, playing a key role in tailoring and personalising a treatment plan according to the phenotype in question. Clinical indications for cardiac MRI are rather similar to those adopted for non-athletic individuals. They can broadly be classified into (i) abnormalities at screening, (ii) positive family history, (iii) grey-zone athletes, (iv) acute cardiac symptoms/presentations, and (v) established cardiovascular disorders.

## 3. Cardiac Magnetic Resonance Imaging in Athletes—A Practical Perspective

### 3.1. Preparation

Preparation for cardiac MRI in athletes is quite similar to the general population. Patient demographics, including age, gender, weight, and height, should be documented on the patient’s scan. Data pertaining to ethnicity and training history are also desirable, as all these variables may potentially impact the quality of the scan, the protocols performed, and reporting at the end. All these characteristics impact athletic cardiac remodelling in different ways. Clinically sound conclusions are a must; inappropriate interpretations may lead to someone being given an erroneous diagnosis.

### 3.2. Expected or Anticipated? Think Twice

MRI sequences and the eventual reporting should be tailored according to these demographic characteristics. Studies show that a minimum of 3 h of training weekly for at least 3 months is sufficient for initial morpho-functional adaptations in the heart [8]. Elite endurance athletes have been shown to have significantly higher absolute and indexed left (LV) and right ventricular (RV) volumes and LV myocardial mass compared to athletes engaging in lower training and sedentary individuals [13]. There is also a strong correlation between VO2_PEAK_, heart rate, and LV/RV volumes [14,15]. Environmental and epigenetic factors may also augment this remodelling process. One in six endurance athletes are genetically predisposed to having bigger ventricles and lower LV/RV EF [16]. A significant proportion of high-performing athletes also exhibit a profound RV remodelling on cardiac MRI, which may raise suspicion of arrhythmogenic right ventricular cardiomyopathy (ARVC), especially when co-existing with other clinical anomalies like symptoms, T-wave inversion, arrhythmias, and replacement fibrosis [17]. The type of remodelling in non-endurance athletes is, however, different. One would expect smaller cavity sizes, a lower myocardial mass, higher ejection fraction (EF), and potentially different patterns of LV geometry [18]. Discordance in the degree of electrical (heart rate during scanning), structural, and functional remodelling in relation to the sporting discipline and training volume may be suggestive of pathology. One should, however, exercise caution in this approach, as high heart rates are also frequently observed in anxious athletes or in those suffering from claustrophobia during the scan.

### 3.3. Technical Challenges—Practical Tips

Anticipating technical challenges whilst scanning will help improve the image quality and reduce the scanning time. Prominent vagal tone with bradycardia and an atrioventricular block, body composition, and a high body surface area may jeopardise the image quality. Bradycardia may lead to long acquisition times, leading to poor-quality cine, parametric mapping, and post-contrast imaging. Increasing segmentation may compensate for long breath holds during scanning [19].

Body composition, with a lower fat content, may also negatively impact the scan quality [20]. A higher volume of distribution (and longer longitudinal recovery during post contrast imaging) may render post-gadolinium imaging non-diagnostic. Longer T1 times, earlier image acquisition, and a higher contrast dose should be considered to compensate for these issues.

A higher body surface area in adults will often manifest as bigger hearts [20]. Image planning should accommodate for this phenomenon, with more slices and/or bigger gaps often required for complete coverage for all four chambers. Younger adolescent athletes with a smaller body surface area, on the other hand, may require thinner slices and smaller interslice gaps. This will inadvertently lead to a shorter scanning time and better patient compliance. Incomplete coverage of ventricles and atria will lead to inaccurate volumetric and functional assessments, potentially rendering a cardiac MRI scan non-diagnostic. Free breathing sequences may also be considered in young children or claustrophobic patients, avoiding the need for general anaesthesia [21].

The pros and cons of the intravenous administration of contrast should also be weighed. Post-contrast imaging may have no role in certain clinical contexts. Serial scanning to assess right ventricular remodelling in congenital heart disease or chamber dimensions and regurgitant volumes in aortic/mitral valve disease are two common scenarios where contrast may not be required.

### 3.4. Contrast Agents

Gadolinium-based contrast agents are typically administered at a concentration of 0.1–0.2 mmol/kg, with image acquisition completed after a delay of 5–20 min. As with any contrast agent, hypersensitivity to such agents is a possibility, although modern agents are well tolerated. The chronic adverse effects of repeated gadolinium administration are, however, well documented, with basal ganglia/nephrogenic systemic fibrosis [22,23]. Gadolinium’s administration is also controversial in pregnancy, which is why it is best avoided. The risk of contrast-induced toxicity is higher in individuals with kidney dysfunction and a low creatinine clearance [24]. One must strike a balance, evaluating the clinical utility and safety of gadolinium-based contrast agents. This risk–benefit assessment is paramount, as post-contrast imaging is essential in risk stratification (cardiomyopathy) and diagnosis (gene carriers, non-dilated left ventricular cardiomyopathy [NDLVC]). Alternative non-gadolinium-based contrast agents are now being proposed, appearing to be safer than their gadolinium counterparts [25].

### 3.5. Cardiac MRI in Athletes with Cardiac Implantable Electronic Devices

Athletes implanted with cardiac implantable electronic devices (CIEDs) frequently pose a technical challenge. Having a CIED was in the past considered to be an absolute contraindication for cardiac MRI. The growing need for cardiac MRI imaging for diagnostic or surveillance purposes has led to significant development in the industry. Most CIEDs are nowadays 1.5 T and 3 T MRI conditional. Several studies have shown that the risk is relatively low [26]. The susceptibility artefact generated by the CIED generator remains a problem from a diagnostic perspective. The artefact may also obscure cardiac chambers, rendering post-contrast imaging non-diagnostic at times. Nevertheless, standardised protocols for CIED may mitigate these issues. One option is to ask athletes with a left-sided CIED implant to raise their ipsilateral arm behind their head during the scan [27]. Opting for a spoiled gradient echo may also improve the image quality. Having a life support team on site, together with ongoing ECG monitoring and physiologists that can interrogate the device, is of paramount importance [26].

### 3.6. Cardiac MRI Protocols

**Figure 1:**
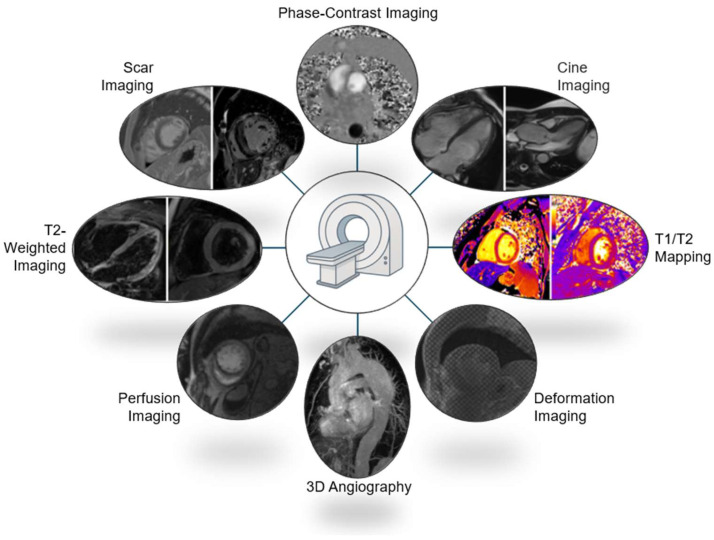
Commonly adopted cardiac MRI sequences when evaluating athletes.

Cardiac MRI protocols in athletic individuals are quite similar to the ones employed for sedentary individuals (Figure 1). Appropriate sequences should be pre-determined according to the clinical indication. Most cardiac MRI protocols will consist of full body scouts, Steady-State Free Precession (SSFP) cine imaging (3 long-axis views and a short-axis stack), phase-contrast flow imaging, parametric mapping, and post-contrast imaging. Different sequences will answer different questions. Scouts are useful to image the great vessels and to evaluate extracardiac shunts in surgically repaired congenital heart disease patients. Cine imaging is required for LV/RV volumetric assessment, EF quantification, and LV/RV wall thickness. Phase contrast is needed to assess valve function and shunt quantification. Mapping sequences are required to rule out interstitial fibrosis, myocardial oedema, and injury. Pre-contrast and post-contrast T1 mapping is required to calculate extracellular volume. Post-gadolinium imaging is required to visualise the extent and pattern of replacement fibrosis [19].

Other dedicated sequences will depend on the clinical indication for the cardiac MRI scan. T2-weighted oedema imaging and T2 mapping a must if there is a suspicion of myocarditis. An axial cine stack of the RV may be required to rule out regional wall motion abnormalities of the RV free wall, with or without fat suppression if there is a suspicion of ARVC or fatty infiltration. A more extended axial cine stack should be considered if anomalous pulmonary venous drainage is suspected. Ischaemia testing (perfusion) with adenosine or regadenoson should be considered if an athlete presents with chest pain in the context of a structurally normal heart or epicardial stenosis. Perfusion imaging is also reasonable when microvascular dysfunction is suspected in athletes with hypertrophic cardiomyopathy (HCM). Tagging sequences to evaluate myocardial deformation are also reasonable in athletes with a suspicion of HCM CMR-feature tracking (CMR-FT) also provides a valuable insight into diseases’ severity and progression, with evidence of subclinical LV and left atrial dysfunction present in a significant proportion of HCM patients with a normal LV EF [28,29]. Early acquisition after gadolinium administration (within 1–2 min) may be required if extensive areas of akinesia raise suspicion of an intracardiac thrombus. Three-dimensional (3D) angiography is useful when there is suspicion of aortic dilatation, aneurysms, or aortic coarctation [19]. The various sequences at the imager’s disposal only serve to improve the diagnostic quality of the scan. Adapting these sequences to the clinical question is paramount for a more personalised treatment pathway.

### 3.7. Gaps in Evidence

Our understanding of the athlete’s heart is constantly evolving. More widespread screening practices, a growing awareness about SCD in athletes, a more permissive approach to sport eligibility, and groundbreaking discoveries in cardiac genetics have generated significant momentum. A paucity of data certainly exists, which makes cardiac MRI reporting all the more challenging. Normal cardiac MRI reference values for athletes is one of the biggest concerns. Large meta-analyses have been published to address this knowledge gap [30]. Authors have devised reference values in different populations. The meta-analysis by D’Ascenzi et al. included 983 male Caucasian athletes, and it looked at LV/RV remodelling and provided mean values for endurance and mixed or combined sports [30]. Data on adolescent athletes and female athletes are now emerging [31,32]. Yet the heterogeneity in the patient populations in most of these studies makes it very hard to generalise the results. The absence of normative data for Afro-Caribbean, Asian, and mixed-race athletes certainly makes cardiac MRI reporting all the more challenging. The implications of unexpected findings like isolated non-ischaemic fibrosis or an isolated high native T1 are also challenging from a more holistic perspective (see the relevant section/s). How would this impact your exercise recommendations, surveillance pattern, and risk stratification? The implications of novel cardiac MRI techniques, like parametric mapping (T1/T2), FT, LV/RV strain, atrial strain, and 4D flow, amongst others, also need to be explored, with a special emphasis on the clinical translation for these modalities [33,34,35].

## 4. Athlete’s Heart

Electrical, structural, and functional changes in athletes may occasionally lead to diagnostic conundrums. Exercise-induced cardiac remodelling is often mild, proportional to training volume and intensity. Significant adaptation can occasionally present diagnostic challenges. Physicians might encounter athletes who appear healthy and asymptomatic but share characteristics with mild forms of cardiomyopathy, a situation often described as the ‘grey zone’. Demographic variables and training history play a key role in the type and degree of adaptation. These characteristics should always be factored in when reviewing athletes referred for advanced cardiac imaging.

Adult and adolescent male athletes often display larger cavity volumes and mass compared to females [32]. Elite endurance athletes also have significantly higher LV volumes and LV mass when compared to non-athletes [13]. Up to 14% of athletes have LV dimensions exceeding 60 mm [36], whilst 11% of Tour de France cyclists showed an LV EF < 52% [37].

Advanced RV remodelling in highly trained athletes is also frequently observed. Up to 16% of trained cyclists may have a reduced RV EF [16]. Up to 40% of endurance athletes have RV outflow dimensions within a range that would be considered diagnostic for arrhythmogenic right ventricular cardiomyopathy [17], with a significant proportion having a borderline low RV fractional area change [38].

LV hypertrophy is certainly the hallmark of HCM, yet the presence of LV hypertrophy is a common clinical manifestation in athletic individuals. Physiological LV hypertrophy is more frequent in males, black athletes, and in those engaging in a moderate-to-high static component (team sports, some endurance sports) [8]. Up to 18% of black athletes exhibit and demonstrate an increased LV wall thickness ≥ 12mm, compared to 4% of white athletes, with a more concentric pattern of hypertrophy in the former group [39]. Male athletes in the grey zone exhibiting an LV wall thickness of 13–16 mm may, however, overlap with HCM [39,40]. The prevalence of LV hypertrophy is far less frequent in females, which is why a more conservative grey zone between 11 and 13 mm has been established in this subgroup [41,42]. Around 2% of white athletes and up to 13% of black athletes are in this grey zone [43]. This is a common occurrence in clinical practice, which is why caution should be exercised when interpreting these scans [43]. The presence of balanced LV hypertrophy and LV dilatation (eccentric hypertrophy) would conversely be more suggestive of athletic remodelling.

Cardiac MRI imaging is an invaluable tool when evaluating athletes in the grey zone. A more precise and in-depth evaluation of cardiac structure can help distinguish ostensibly normal healthy athletes from those harbouring early cardiomyopathy. It is especially invaluable in establishing LV and RV volumes, RV inflow and outflow tracts, and the LV wall thickness and apex. Sequences targeting tissue characterisation may also help rule out early disease, including ischaemia, oedema, and interstitial or replacement fibrosis [44].

## 5. Family History of Cardiomyopathy

A family history of cardiomyopathy is a valid reason for cardiac MRI in athletes. Abnormal ECGs displaying pathological ECG anomalies like a left bundle branch block or T-wave inversion will require a cardiac MRI to rule out cardiomyopathy, even in the presence of a normal heart on echocardiography [8]. One may also consider a cardiac MRI in those athletes with sinister symptoms, even in the absence of abnormalities on ECG or echocardiography. This is especially relevant in the case of malignant pro-arrhythmic genes that are associated with fibrosis, even in the absence of chamber dilatation or dysfunction [10].

Referral for cardiac MRI may also be justified in athletes who report a family history of SCD, especially in those cases where cardiomyopathy was the presumed cause of death at autopsy. Athletes who are gene-positive for the familial disease-causing variant should also be offered cardiac MRI [9,10]. Concealed cardiomyopathy in the absence of echocardiographic observation is typical of certain sarcomeric mutations like Filamin C (FLNC) or Desmoplakin (DSP) [9]. NDLVC presenting with a non-ischaemic scar would otherwise be missed (see relevant section).

## 6. Pre-Clinical Cardiomyopathy

Structural abnormalities are a prerequisite for a cardiomyopathy diagnosis, with different phenotypes having specific diagnostic criteria. The role of cardiac MRI in athletes with cardiomyopathy is unparalleled, yet one should not underestimate the potential of this imaging modality in cases where a cardiomyopathy diagnosis may not be overtly apparent. The presence of certain pathological ECG anomalies, like T-wave inversion, Q waves, left bundle branch block, low QRS voltages, and ventricular arrhythmias, is sometimes observed when screening athletic individuals [45,46,47,48,49,50,51,52,53]. These anomalies will invariably be associated with pathology, which is why a comprehensive evaluation with cardiac MRI is warranted. The diagnostic yield with cardiac MRI is often low with these ECG patterns, especially when echocardiography is normal.

**Figure 2:**
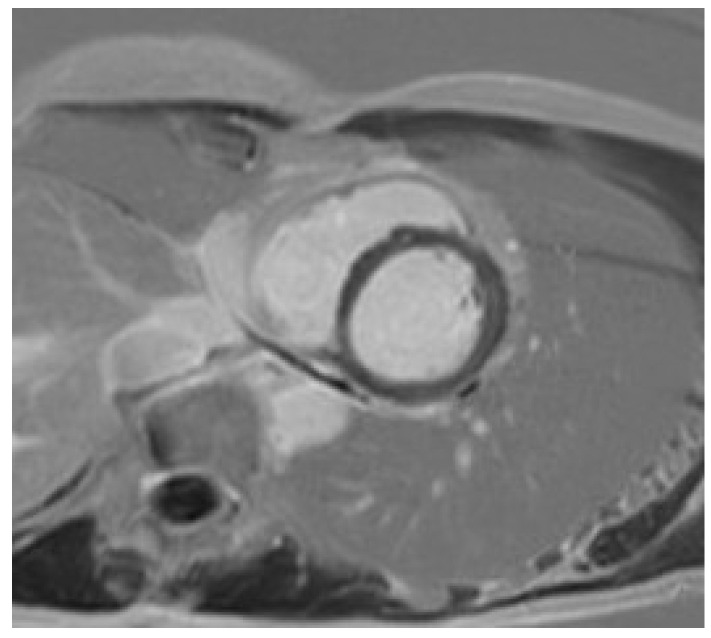
A mother presents to clinic for screening following the sudden death of her son who was a long-distance runner. Autopsy confirmed hypertrophic cardiomyopathy (HCM) secondary to a pathogenic TNNT2 variant. The victim’s uncle was also diagnosed with HCM in the interim and referred for transplantation. The mother was a carrier for the TNNT2 variant. The ECG was abnormal; echocardiography was normal. CMR identified mid-wall fibrosis in the lateral wall, despite the absence of left ventricular hypertrophy.

Certain subtle structural abnormalities (focal LV hypertrophy [54], mitral valve leaflet abnormalities [55], abnormal papillary muscle morphology and insertion [55], myocardial crypts [56]), which may be suggestive of early cardiomyopathy are easily missed with an echocardiography-only approach. The presence of interstitial and replacement fibrosis is also only established with cardiac MRI (Figure 2). This is especially important in athletes with an abnormal ECG who have a family history of NDLVC. The presence of a high T1 on a pre-contrast mapping sequence may be suggestive of extracellular volume (ECV) expansion and early cardiomyopathy. A high T1 should however always be interpreted in the clinical context; this finding when present in isolation is often of no clinical relevance [12]. Focal or diffuse replacement fibrosis in the absence of ventricular dilatation or dysfunction is also typical of certain genetic variants like FLNC, DSP, or Plakophillin-2 [PKP2]).

## 7. Hypertrophic Cardiomyopathy

HCM is a common inherited cardiac condition, affecting 1 in 200 to 1 in 500 individuals [57]. The 12-lead ECG is abnormal in 75–95% of patients with HCM [58]. hence why it is one of the commonest disorders identified at screening. The definition of HCM has evolved over the past decade, with genetic testing and cardiac MRI identifying more subtle HCM phenotypes that would have been missed with echocardiography. The phenotypic traits of HCM extend beyond LV hypertrophy. Most patients have other structural manifestations of the disease [55], including (a) LV trabeculation [59], (b) LV aneurysms [55], (c) mitral valve leaflet elongation [55], (d) abnormal papillary muscle anatomy, (e) LV trabeculation [60], (f) abnormal septal convexity [61], (g) perfusion defects [62], and (h) non-ischaemic fibrosis. Some of these structural anomalies may also present early on in the disease, with myocardial crypts, mitral valve anomalies, LV trabeculation, and perfusion defects often preceding LV hypertrophy [55,60,62].

Athletes with HCM undergoing cardiac MRI are often subjected to various sequences that each target specific features of the disease. Cine imaging accurately delineates the LV endocardial and epicardial borders, enabling accurate assessment of LV wall thickness, chamber size and function, myocardial crypts, aneurysm formation, and mitral valve abnormalities. Phase-contrast flow imaging is used to assess the degree and mechanism of LVOT obstruction. Tagging sequences are used to evaluate myocardial deformation. CMR-FT could also identify subclinical myocardial dysfunction even in the absence of LV hypertrophy [63]. Its role in evaluating athletes in the grey zone has, however, not yet been explored. T1 mapping and post-contrast imaging (LGE) play a key role at ruling out the presence and extent of interstitial and replacement fibrosis.

LV hypertrophy is a frequent physiological manifestation in athletes, some potentially overlapping with early HCM. An abnormal LV geometry or morphologically supporting features should raise suspicion for HCM. Athletes with HCM often exhibit an asymmetric pattern of LV hypertrophy, with an apical HCM variant present in 36% of cases [64]. Concentric LV remodelling or hypertrophy in athletes is also infrequent [18]. Certainly, the presence of an abnormal LV geometry [18] and concomitant clinical features (a family history of HCM, sarcomeric mutation, abnormal ECG) in the presence or absence of structural anomalies (small LV cavities, MV abnormalities, small aneurysms, non-ischaemic fibrosis) should raise suspicion of pathological LV hypertrophy.

Severe HCM phenotypes are often incompatible with vigorous physical activity or competitive sports, which is why athletes with HCM often exhibit less obvious imaging features of the disease. They have milder degrees of LV hypertrophy when compared to sedentary HCM patients, with a predilection for apical thickening [64].

Technically challenging echocardiography studies in slim athletes also make it hard to visualise the apex, easily missing out on relative apical hypertrophy. Segmental LV hypertrophy (inferior/lateral wall), LV aneurysms, and mitral valve abnormalities may also go unnoticed on echocardiography [65,66]. The high spatial resolution with cardiac MRI helps to overcome these challenges, obtaining a better and more reproducible definition of LV wall thickness.

**Figure 3:**
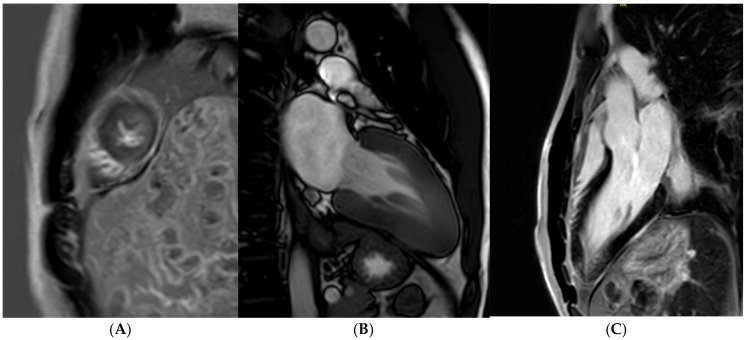
Caucasian male soccer player presenting with inferolateral T-wave inversion on ECG. SSFP cine imaging (**A**) showing apical hypertrophy, with evidence of apical fibrosis on post-contrast imaging (**B**,**C**).

Tissue characterisation with cardiac MRI also plays a key role in athletes with a suspicion of HCM. Post-contrast imaging with late-gadolinium enhancement (LGE) is used to detect macroscopic replacement fibrosis (Figure 3). The presence of fibrosis is diagnostic of a disease process, often patchy and present in hypertrophied segments. Extensive LGE has also been associated with an increased risk of ventricular arrhythmias and SCD [67,68,69]. LGE is, however, only present in 60% of HCM cases [68,70], implying that the absence of fibrosis should not preclude labelling an athlete with HCM. T1 mapping has an important role in LGE-negative HCM cases. Myocyte hypertrophy, rather than expansion of the extracellular matrix, often leads to lower T1 values, especially in endurance and male athletes. This provides a valuable insight into the pathophysiological process of athletes with LV hypertrophy [71,72,73]. Pathological LVH, on the other hand, is positively correlated with ECV, suggesting that an increase in the extracellular matrix is the main contributing factor towards an elevated LV mass [74]. T1 mapping certainly holds promise, yet it requires extensive validation in large athletic cohorts with HCM. LGE and mapping sequences may also play a diagnostic role in HCM phenocopies when cardiomyopathy coincides with extracardiac manifestations. There is a typical abnormal post-contrast myocardial nulling in cardiac amyloidosis. T1 values are, conversely, significantly reduced in Fabry’s disease [75].

**Figure 4:**
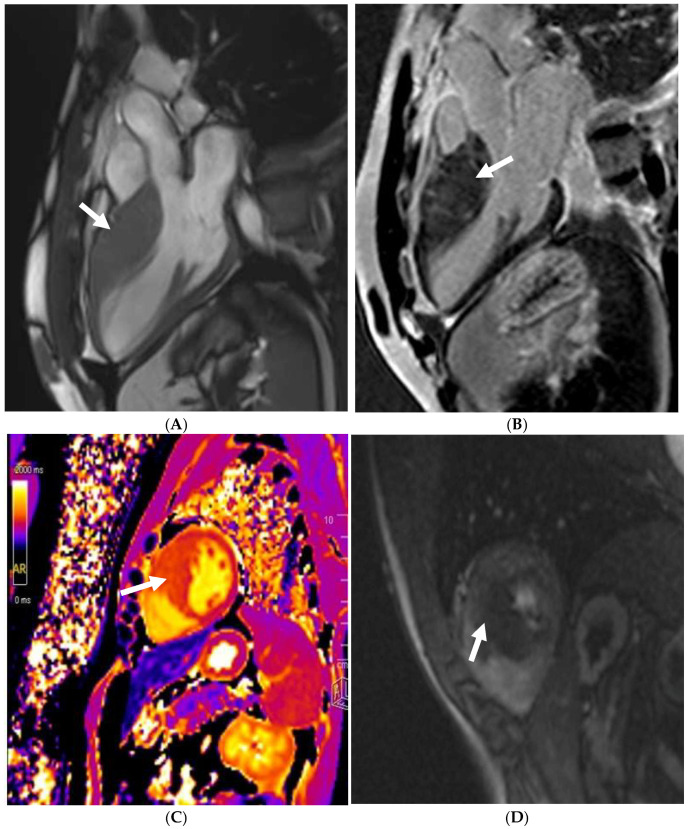
A 21 year old male that presented with chest pain during a pre-participation evaluation to join the armed forces. ECG showing biphasic ST segments in leads V1-V3, with T-wave inversion in II/III/aVF. CT coronary angiogram ruled out epicardial coronary disease. A stress echocardiogram ruled out dynamic LVOT obstruction. Echocardiogram confirmed the presence of hypertrophic cardiomyopathy, secondary to a likely pathogenic MYBPC3 variant. Cardiac MRI (3T scanner) showing septal hypertrophy (arrow) (**A**), extensive replacement (arrow) (**B**), and interstitial fibrosis in the septal segments (arrow) (**C**) (T1 map using a MOLLI 5b(3b)3b [3T]). Perfusion imaging (**D**) also identifying an extensive perfusion defect (arrow) in the hypertrophied segments, confirming microvascular dysfunction.

Small-vessel disease and microvascular dysfunction is a phenotypic trait in some HCM patients, often leading to a demand and supply perfusion mismatch in HCM patients (Figure 4) [58,76]. One small study also confirmed that coronary vasodilation reserve is preserved in athletes with physiological LV hypertrophy but reduced in patients with HCM [77], suggesting it may play a role when evaluating athletes in the grey zone. Data supporting routine ischaemia testing in athletes with HCM are inexistent. For HCM athletes who report chest pain without a documented LVOT obstruction (either at rest or during exercise) and epicardial coronary disease, our current practice is to perform perfusion imaging to specifically rule out microvascular dysfunction.

## 8. Dilated Cardiomyopathy

Dilated cardiomyopathy (DCM) is a non-ischaemic myocardial disorder that is defined as the presence of unexplained LV chamber dilatation, often in combination with global or regional systolic dysfunction. Right ventricular dilatation, dysfunction, or regional wall motion abnormalities may also co-exist with left-sided disease. DCM is estimated to effect 1 in 250 to 1 in 500 individuals in the general population. It is a rare cause of sudden death in athletic individuals, contributing to around 1% of all deaths in athletic cohorts [7].

The presence of LV dilatation may at times pose a diagnostic challenge when evaluating athletes. As described previously, biventricular dilatation is often present in athletic individuals, with a predilection for endurance and male athletes. This is especially true when co-existing with a preserved LV EF or in cases with an only mildly reduced systolic function. SSFP cine imaging (increasing segmentation in bradycardic individuals for a better image quality) are the cardiac MRI sequences required for volumetric analysis.

**Figure 5:**
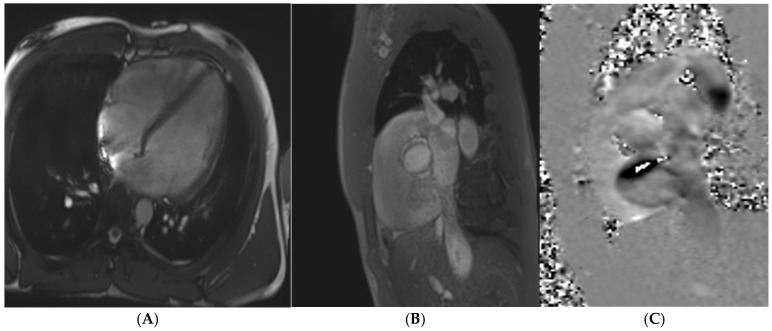
A 21 year old Caucasian male found to have lateral T-wave inversion on pre-recruitment ECG to join the military. Echocardiography showed dilated left and right ventricles, a bicuspid aortic valve (AoV), and possibly an atrial septal defect (ASD). Cardiac MRI confirmed the presence of dilated ventricles (**A**) exceeding reference ranges for male athletes. A bicuspid AoV (Sievers Classification Type 0) was also confirmed (**B**), without regurgitation. Phase-contrast flow imaging confirmed the presence of an ASD (**C**). Post-contrast imaging showed no replacement fibrosis.

Cardiac MRI plays a key role in the diagnostic evaluation of athletes in the ‘grey-zone’. Cardiac MRI is the gold standard for evaluating chamber dimensions and global and regional systolic function of both ventricles [78]. The degree of ventricular dilatation is also helpful from a diagnostic perspective. A very dilated LV > 60mm in end-diastolic (LVIDd) is only present in 15% of elite athletes [36], dropping to <1% if exceeding 70mm [43]. Cardiac MRI reference ranges specific to athletes, derived from meta-analysis, can contribute to identifying individuals with extreme remodelling patterns [30]. The relationship between the LV and RV can also help discriminate between a physiologically vs. pathologically dilated ventricle (Figure 5). Proportionally dilated ventricles favour athletic remodelling, provided this tallies with the demographic and training profile of the athlete.

Tissue characterisation with LGE imaging and T1 mapping offers a more comprehensive evaluation compared to echocardiography. The presence of non-ischaemic fibrosis in a subepicardial or mid-wall distribution is diagnostic of a pathological process, effectively ruling out athletic remodelling. The absence of myocardial fibrosis is, however, a reality in most DCM patients, with LGE only present in 40% of DCM patients, with a predilection for the septum [79]. The presence, extent, and pattern of fibrosis offers significant prognostic information. Minor focal myocardial fibrosis in the RV insertion points is frequently observed in athletes, reported in up to 69% [80]. This is not a marker of disease but is thought to occur in response to chronic RV pressure and/or volume overload during exercise [81]. Septal LGE, in contrast, has been shown to be heavily linked to an increased risk of ventricular arrhythmias and SCD, especially when in combination with free-wall LGE [79]. Replacement fibrosis is irreversible and may actually progress in a subset of patients [82]. The presence of a ring-like LGE distribution, defined as LGE in ≥2 contiguous LV segments, has also been shown to carry prognostic value for arrhythmia and heart failure severity [83,84].

The extent and pattern of fibrosis may also be useful to determine the aetiology of LV dysfunction. The presence of a subepicardial or mid-wall LGE in the inferolateral wall may be suggestive of a past myocarditis. A patchier distribution may raise the suspicion for sarcoidosis, especially when there is concomitant RV involvement. Septal mid-wall LGE is typical of LMNA patients. Ring-like mid-wall or subepicardial LGE is suspicious for Desmoplakin (DSP) or Filamin C (FLNC) cardiomyopathies. Early referral for genetic testing, in combination with a rigorous risk assessment, in athletes with these sinister phenotypes is encouraged [75]. As part of the shared decision-making approach, discussions about an implantable cardioverter–defibrillator and exercise restriction may be required in a subset of athletes [9].

Other non-conventional or novel techniques may positively impact the diagnostic utility of cardiac MRI. Parametric mapping appears promising, especially when evaluating phenotype-negative, gene-positive carriers (see pre-clinical cardiomyopathy section). It also plays a role in athletes with chamber dilatation without replacement fibrosis. CMR-FT also holds some promise in this regard [34].

## 9. Non-Dilated Left Ventricular Cardiomyopathy

The NDLVC phenotype is a novel concept proposed in the latest cardiomyopathy guidelines, where patients were inherently being given a diagnosis of DCM, despite the absence of LV dilatation. This new umbrella term also includes those individuals labelled with arrhythmogenic left ventricular cardiomyopathy [75]. The prevalence of this new disease entity remains to be established.

**Figure 6:**
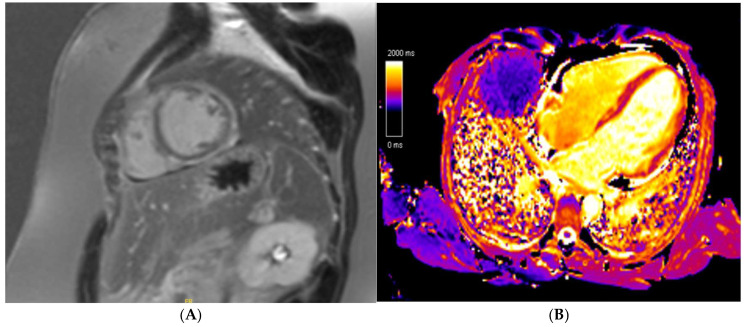
A 21 year old endurance athlete, referred for screening as her mother was diagnosed with arrhythmogenic cardiomyopathy secondary to a Desmoplakin pathogenic variant. The athlete was also gene-positive, with a normal ECG and echocardiogram. Cardiac MRI identified a ring-like subepicardial scar on post-contrast imaging (**A**), with a diffusely elevated T1 (**B**), resulting in a diagnosis of non-dilated left ventricular cardiomyopathy.

Non-ischaemic replacement fibrosis or fatty replacement in the presence or absence of LV dysfunction (LV EF < 50%) or regional wall motion abnormalities constitute the main imaging findings required for an NDLVC diagnosis. Cine imaging, T1 mapping, and LGE imaging should be performed if there is suspicion of NDLVC. The presence of LGE in NDLVC is why cardiac MRI is key when evaluating athletes with NDLVC. Similar to DCM, an inflammatory (acute or chronic myocarditis) or genetic component (DSP, FLNC, Desmin [DES], Phospholamban [PLN], LMNA) are the main drivers towards myocardial fibrosis in NDLVC [75]. The extent and degree of scarring again offers substantial prognostic information, similar to the DCM phenotype (Figure 6).

The NDLVC may also act as a precursor to DCM. The presence of non-ischaemic fibrosis predating LV dilatation and/or dysfunction is well established [85]. Unexplained ventricular arrhythmias may also be the manifestation of early concealed cardiomyopathy [86], hence why cardiac MRI to rule out scarring is pivotal in these patients.

Isolated focal (one or two segments) non-ischaemic LV LGE in athletes is an area of ongoing interest. The prevalence has been reported in up to 50% in some studies [87,88,89]. This phenomenon appears to be associated with ventricular arrhythmias and sudden death in athletes [90]. A recent study also showed that 50% of male endurance master athletes had non-ischaemic fibrosis, often confined to the basal lateral LV wall [91]. Very limited data on female athletes in this regard are currently available. The clinical evaluation should be adjudicated on a case-by-case basis.

## 10. Arrhythmogenic Right Ventricular Cardiomyopathy

ARVC is an important disease entity in athletes, accounting for 4–22% of all SCDs [43]. It is characterised by myocardial atrophy with fibro-fatty replacement in the RV, affecting males more frequently than women. Patients with ARVC often have variants encoding cardiac desmosome proteins. PKP2 is the most common, accounting for 50% of cases. Other variants include Desmoglein-2 (DSG2), Desmocollin-2 (DSC2), Plakoglobin (PG), PKP2, and DSP [75]. A significant proportion remain gene-elusive. A comprehensive imaging assessment that should be followed up with genetic testing is key; the heightened risk of a cardiac arrest and disease acceleration caused by PKP-2-mediated ARVC is particularly relevant for athletes [9,92,93,94].

Cardiac MRI plays a key diagnostic role, contributing towards major and minor criteria in the ‘updated ARVC criteria’ published by the Heart Rhythm Society [95]. The major criteria are dependent on RV chamber size (RV end-diastolic volume ≥ 110mL/m^2^ in males or ≥100mL/m^2^ in females), RV EF (<40%), and RV regional wall motion abnormalities (hypokinesia, dyskinesia, or akinesia). The minor criteria are dependent on the same variables, though requiring lower indexed RV volumes (>100 mL/m^2^ in men, >90 mL/m^2^ in females).

Healthy endurance athletes in the grey zone may invariably overlap with those athletes having an established ARVC [17]. Cardiac MRI is a superior modality to echocardiography for accurately assessing RV volume, function, and regional wall motion abnormalities. Up to 40% of athletes satisfy RV outflow dimensions compatible with an ARVC diagnosis [17], whilst some have a borderline reduced RV function [38]. Balanced RV and LV dilatation has been shown to support athletic remodelling, though RV:LV ratios have been proposed as markers of ARVC [17,96]. Cardiac MRI is also superior at detecting regional wall motion abnormalities. These are easily missed on echocardiography, yet are highly specific to ARVC, even in the absence of RV dilatation or dysfunction [17,96]. Genetic susceptibility may also contribute to RV dilatation and a reduced RV EF in a subgroup of athletes [16].

**Figure 7:**
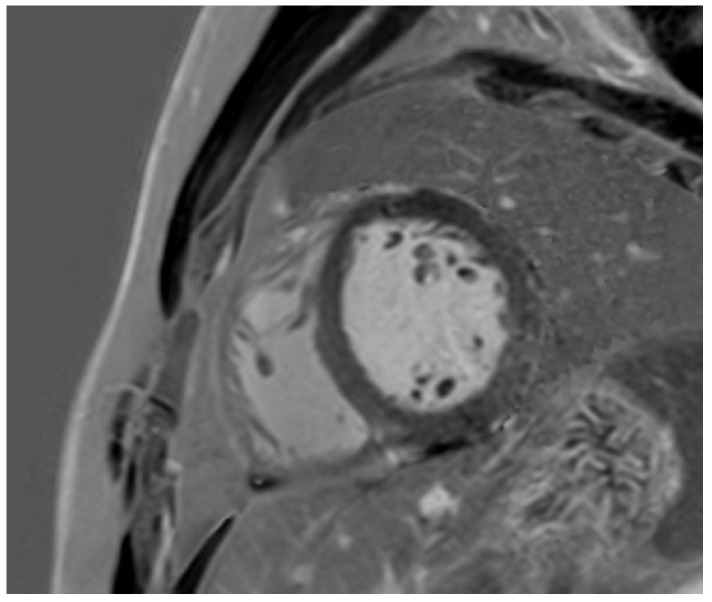
An 18 year old male Caucasian competitive swimmer who was seen because of a very long PR interval in the context of non-cardiogenic syncope. Echocardiography revealed a dilated LV. This was confirmed on CMR, though perfectly within normal athletic specific reference ranges. Post-contrast imaging showed posterior RV insertion-point fibrosis.

Cine SSFP imaging plays a key role in determining RV volume and function. An axial stack of the whole heart, with a reasonably small interslice gap, may also help identify subtle regional wall motion abnormalities in the RV free wall. LGE imaging has been incorporated in the proposed Padua Criteria when there is a suspicion of biventricular arrhythmogenic cardiomyopathy (see previous section) [97]. LGE to rule out RV scarring is, however, often limited in efficacy, as the fibrosis is often very subtle due to the thin-walled RV wall. As discussed previously, RV insertion point fibrosis is frequently observed in athletes and is not a marker of disease (Figure 7).

## 11. Left Ventricular Hypertrabeculation

**Figure 8:**
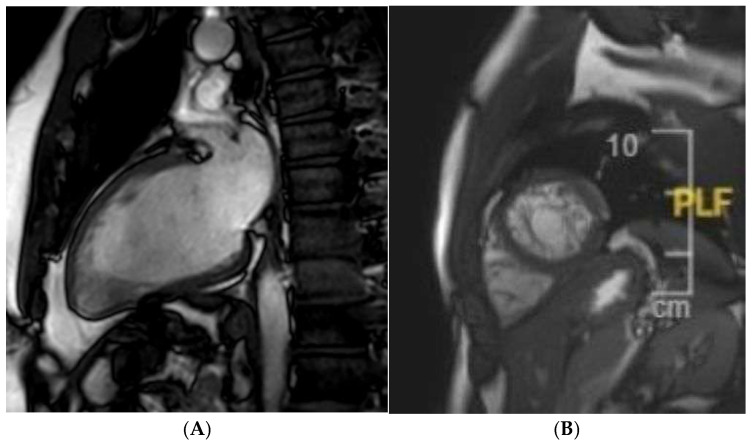
A 19 year old male soccer player who presented with dyspnoea, found to have increased LV trabeculation on echocardiogram. Cardiac MRI showed prominent apical hypertrabeculation, with preserved thickness of the non-compacted layer. There was preserved LV function, with a normal LV size and absence of regional wall motion abnormalities and fibrosis. The findings were attributed to athletic remodelling.

LV hypertrabeculation, formerly referred to LV non-compaction, is no longer a pathological entity but is now considered a physiological epiphenomenon [75]. It is characterised by prominent LV trabeculae and deep myocardial recesses, often present in isolation. Up to 8.1% of athletes fulfil the previously established LVNC criteria [98], and it is more prevalent in Afro-Caribbean individuals and in males (Figure 8). This high prevalence confirms that this is a physiological trait when present in isolation. It has also been suggested that extreme forms of LV trabeculation may contribute towards better rotation and twisting of the apex, improving the mechanical efficiency of the heart during systole and diastole [99].

However, this phenotypic trait may co-exist with HCM and DCM, often coinciding with LV wall thinning, LV dysfunction and/or dilatation, arrhythmias, and thromboembolic phenomena. Cardiac MRI is helpful to rule out pathological features that may suggest an underlying cardiomyopathy. Endocardial border definition allows for better visualisation of the recesses. Areas of myocardial thinning or regional wall motion abnormalities are also better appreciated. Early-gadolinium enhancement (EGE) may be useful to determine the presence of thrombi in the recesses, whilst LGE imaging is useful to rule out replacement fibrosis. Ruling out additional structural manifestations is more important if athletes present with symptoms like an abnormal ECG or LV dysfunction or in the context of a family history of cardiomyopathy or SCD.

## 12. Mitral Valve Prolapse

Mitral valve prolapse (MVP) is a common valvular anomaly, with an estimated incidence of 2–3% in athletes and non-athletes [100,101]. MVP is an important cause of SCD in athletes, with an estimated annual SCD rate of 0.4–1.9% among patients with MVP [102]. The prevalence of MVP goes up to 11.7% in patients with unexplained SCD [103]. Most MVPs confer a low risk of SCD, with risks predominantly linked to haemodynamically significant mitral regurgitation (MR) and adverse cardiac remodelling. The malignant MVP phenotype is, however, a peculiar phenotype linked to malignant ventricular arrhythmias even in the absence of significant MR [102]. MVP in the presence of a family history of SCD, arrhythmias, inferior T-wave inversion and QT prolongation on ECG, mitral annulus disjunction (MAD), and significant MR should raise suspicion of the malignant MVP phenotype. Cardiac MRI plays a key role in quantifying MR, whilst also ruling out basal inferolateral wall fibrosis when co-existing with other high-risk features [9,104].

**Figure 9:**
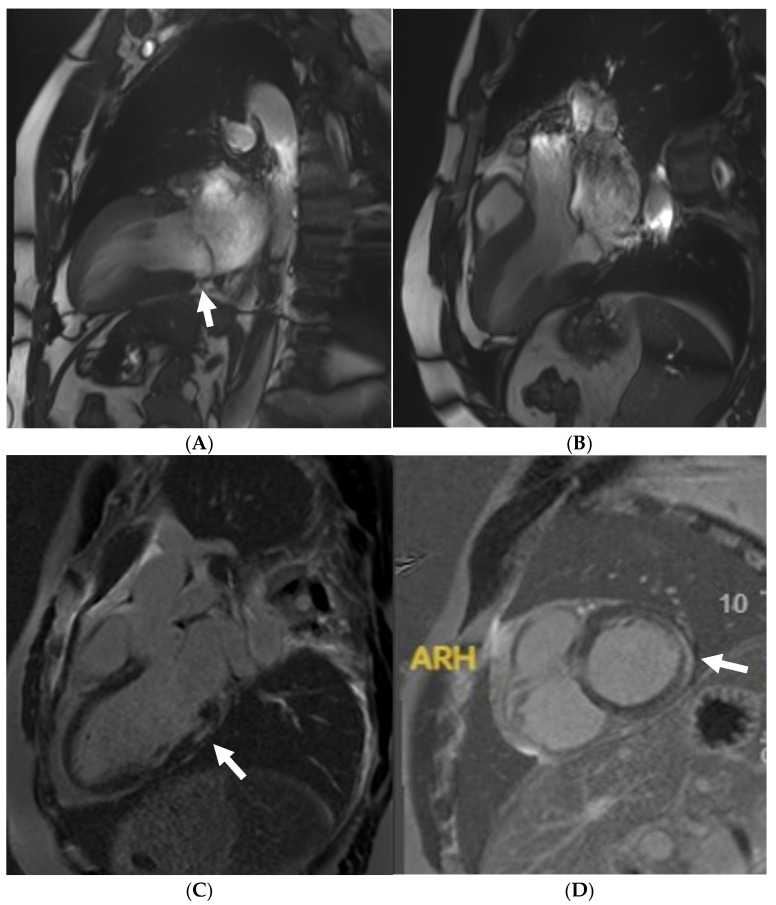
A 42 year old Caucasian male, engaging in power sport (recreational) for 20 years, presented with a profound drop in exercise tolerance. Murmur heard on physical examination. T-wave inversion in the inferior leads noted. Supraventricular arrhythmias during an exercise test. Mitral valve (MV) prolapse with moderate regurgitation noted on echocardiogram. Cardiac MRI showed a dilated LV, an LV ejection fraction of 58%, and mitral annular disjunction (arrow) (**A**). There was prolapse of the posterior MV leaflet with moderate regurgitation (**B**). Post-contrast imaging identified a subepicardial scar in the lateral wall (arrow) (**C**,**D**), consistent with a diagnosis of malignant MV prolapse. He was subsequently referred for MV surgery.

The cardiac MRI acquisition should include standard long-axis and short-axis cine stacks, phase-contrast sequences of the aortic and pulmonary root, and post-contrast sequences [105,106]. These sequences provide a comprehensive morpho-functional evaluation of the mitral valve and subvalvular apparatus, including localising prolapsing segment/s, prolapse severity, leaflet thickness, papillary muscle anatomy, and MAD and MR quantification. CMR has been found to be more sensitive to identifying MAD compared to transoesophageal echocardiography and transthoracic echocardiography [107]. Additionally, cardiac MRI aids in identifying anterior MVP, as it directly correlates with non-ischaemic fibrosis of the papillary muscles and LV inferolateral basal segment (Figure 9). Both of these are linked to prolapsed leaflets and annulus hypermobility [108]. In cases of minimal MR, cardiac MRI may reveal a disproportionate LV remodelling and fibrosis associated with malignant arrhythmias, facilitating the identification of athletes at elevated risk [104]. Further, there is emerging evidence of the role of cardiac MRI in identifying myocardial ECV through T1 mapping. Studies have demonstrated that ECV is increased in MVP patients with MAD compared to those without MAD and MR or isolated MVP [108]. Moreover, MVP patients who have sustained out-of-hospital cardiac arrest exhibit a higher ECV and LGE [30].

Athletes with MVP in the absence of high-risk features are often cleared for competition. The presence of any of the high-risk features should prompt referral to sport cardiology experts, with cases adjudicated on a case-by-case basis in a shared-decision approach [9,10].

## 13. Bicuspid Aortic Valve and Aortopathy

Bicuspid aortic valve (BAV) is the most common congenital heart disorder, with an estimated prevalence of 1 to 2% [109]. Echocardiography remains the primary imaging modality for diagnosis [110,111]. However, cardiac MRI offers additional diagnostic value beyond echocardiographic parameters [112].

**Figure 10:**
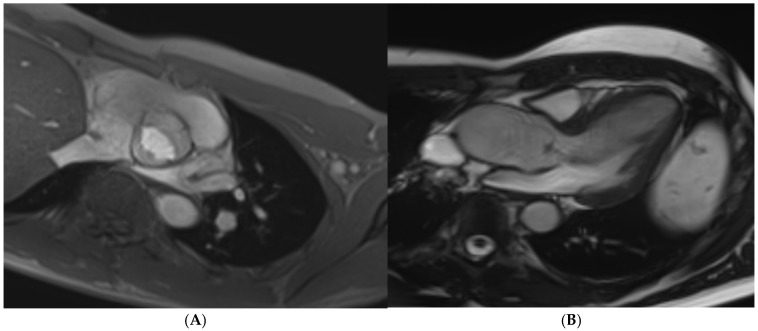
A 24 year old long-distance runner reported a drop in exercise tolerance. A murmur was heard at auscultation. Cardiac MRI showed a bicuspid aortic valve (Sievers Classification Type 0) (**A**), with eccentric closure and moderate regurgitation (**B**).

Cine imaging with balanced SSFP cine sequences and phase-contrast flow imaging facilitate a morpho-functional assessment of the aortic valve (AV) (Figure 10), including the number of raphes and cusps (Sievers Classification), cusp motion, and the quantification of aortic valve insufficiency (AI) and stenosis (AS) [111]. Cardiac MRI also allows for the accurate measurement of LV volumes, playing a key role in discerning training-related LV remodelling and pathological volume overload from AI. In such cases, athlete-specific LV reference ranges should be applied for the correct interpretation [30,31,32,113]. Myocardial fibrosis detected by a high native T1 and the presence of LGE on cardiac MRI are considered poor prognostic markers. This in-depth phenotyping could lead to a more personalised risk assessment in athletes with BAV [114,115].

Cardiac MRI in athletes with BAV is also useful at ruling out other concomitant congenital anomalies, including ventricular septal defects and aortic pathology [116]. Aortic abnormalities in BAV vary and may include coarctation, aortic dilatation, patent ductus arteriosus, and an interrupted aortic arch [111,117]. Aortic disease may also exist in the absence of BAV, broadly classified as hereditable thoracic aortic disease (HTAD), acquired or unexplained. Acute aortic syndrome (AAS) is a rare cause of SCD in athletes [118]. Aortic calibre, the nature of the aortopathy, and sports discipline appear to be risk factors for AAS [119]. Reference data for aortic dimensions in athletes are limited and diverse; a marked enlargement exceeding 42 mm in male athletes and 40 mm in female athletes requires further evaluation and surveillance [9].

**Figure 11:**
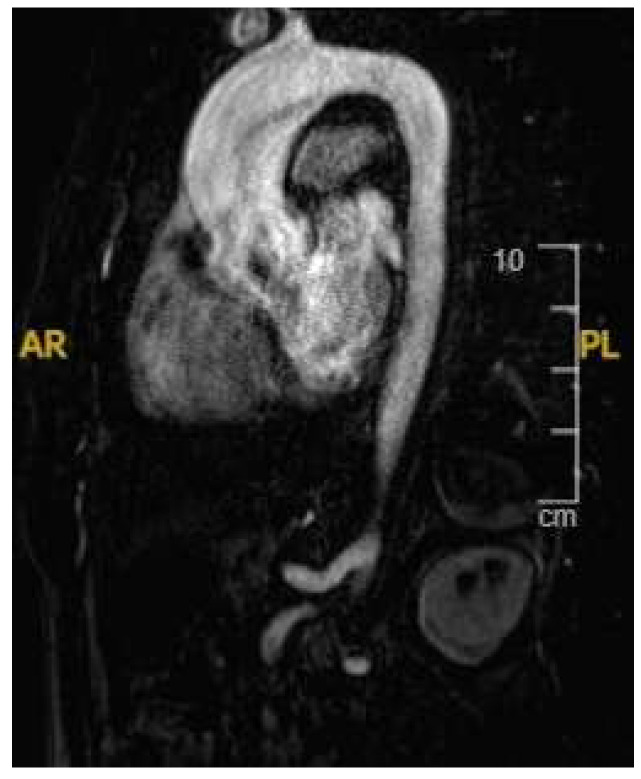
In a 34 year old Caucasian male soccer player, a routine screening echocardiogram identified a dilated proximal ascending aorta. He had a normal blood pressure; genetics was negative. A 3D aortogram on cardiac MRI confirmed the presence of a dilated aortic root and proximal ascending aorta, measuring 43 mm.

Contrast-enhanced magnetic resonance angiography (MRA) and three-dimensional SSFP sequences provide a precise visualisation of the aorta, enabling accurate measurements and the detection of associated aortic anomalies (Figure 11) [117,120]. Aortic dimensions should be measured as recommended by the guidelines, comparing serial scans side by side whenever possible. Repeat imaging in 6 to 12 months to evaluate the disease’s progression is recommended, which will help establish a tailored surveillance strategy depending on the rate of progression and severity of the condition [9,121]. Cardiac MRI is particularly advantageous for long-term monitoring compared to computed tomography (CT), as it avoids repeated contrast exposure. This is especially relevant for athletes, given their predisposition to aortic root remodelling due to the repeated haemodynamic overload from exercise training, necessitating serial imaging over years [11,122]. More recently, four-dimensional (4D) flow imaging has emerged as a valuable tool for evaluating the mechanism of BAV-associated aortic dilation and remodelling by assessing wall shear stress and flow patterns [123,124].

Exercise recommendations for athletes with BAV follow the same guidelines as those with a tricuspid aortic valve in the absence of aortopathy (discussed further in the next section). Recent evidence suggests that athletes with BAV behave similarly to non-athletes with BAV, though admittedly the longitudinal impact of exercise on valve function remains to be determined [125]. A personalised approach is advocated in athletes with moderate to severe AI and stenosis, especially when coinciding with other congenital anomalies and/or aortic dilatation. This approach also extends to those athletes with aortopathy in the absence of BAV. Current guidelines recommend avoiding competitive sports if the aortic dimensions exceed 45 mm; this is especially relevant in those engaging in power sports [9]. Athletes with unexplained thoracic aneurysms are also discouraged against competitive sport, especially if their aortic dimensions exceed 44 mm. The risk of complications is higher in these athletes, which is why a full aorta assessment with CMR is paramount [9,121]. A more permissive approach is now advocated for those athletes with an aortic calibre of 40–44 mm, provided there are no additional risk factors like a family history of aortic dissection or rapid aortic progressive dilatation (≥3 mm per year) [9]. This shared decision-making approach is especially relevant in athletes with HTAD and normal aortic calibres. Exercise testing with blood pressure monitoring should complement CMR imaging in the evaluation of these athletes [9].

## 14. Other Valvular Heart Disease

Valvular heart disease (VHD) affects 1–2% of young individuals in the general population who are engaged in regular exercise [10]. While degenerative VHD is more common in older athletes, younger athletes may have congenital-related VHD. Among athletes, mild regurgitant valves are considered physiologically normal variants as part of the athlete’s heart [10].

The European Society of Cardiology recommends the use of cardiac MRI in patients with poor echocardiographic windows, particularly for assessing regurgitant valvular lesions. Cardiac MRI also offers additional value in VHD by evaluating biventricular volume and function, quantifying peak velocity and regurgitant volumes, and detecting associated aortic abnormalities which may contribute towards surgical decision-making [11,126,127]. Notably, cardiac MRI is widely recognised as the gold standard for RV assessment, making it particularly useful in cases involving tricuspid and pulmonary valve disease [126,128,129]. It may also be used to assess myocardial fibrosis, which has been shown to provide prognostic information [130].

B-SSFP or spoiled gradient echo sequences (which are more sensitive to flow turbulence) may be used for valvular motion imaging and direct planimetry in stenosed valves; phase-contrast velocity mapping allows for the direct quantification of regurgitant volumes and fractions, as well as velocities across stenotic valves, and LGE imaging is used to assess fibrosis [131,132].

Athletes with mild to moderate valvular lesions may continue sports if they remain asymptomatic and do not exhibit any negative prognostic factors such as ventricular dysfunction or a reduced functional capacity. However, patients with severe valvular disease should be referred to expert centres for advice [9,10,11,126].

## 15. Congenital Heart Disease

The prevalence of all types of congenital heart disease (CHD) ranges from 2 to 9 per 1000 live births. There is substantial geographic variation, partly due to improved surgical techniques and more advanced medical care [133]. As more CHD patients survive into adulthood, sports cardiologists must be well versed in evaluating eligibility for competitive sports participation, with CMR playing a crucial role. CMR remains the gold standard for volume estimations, particularly for RV assessment [128,134]. Apart from this, cardiac MRI provides critical insights into shunt quantification, pulmonary valve abnormalities, and myocardial fibrosis, which has prognostic and therapeutic implications in adult CHD patients [135,136,137].

**Figure 12:**
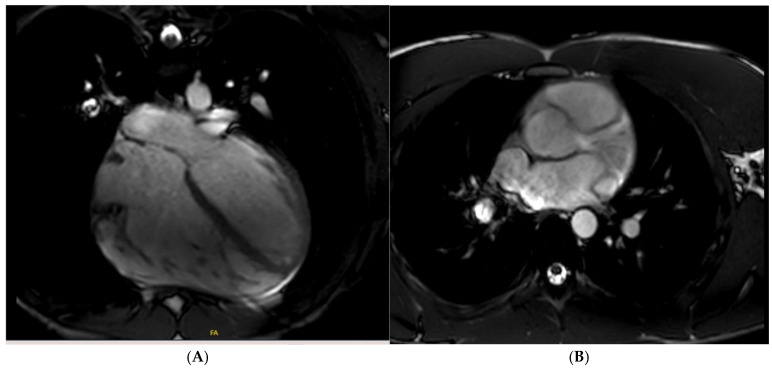
A 22 year old Caucasian male soccer player who presented for a routine screening echocardiogram. A dilated right ventricle (RV) led to a cardiac MRI. This confirmed a dilated RV (**A**), exceeding athletic reference ranges. MRI also identified a sinus venosus defect and anomalous pulmonary venous drainage (**B**), with a Qp:Qs > 1.5, reaching surgical thresholds.

Cardiac MRI sequences for RV assessment include b-SSFP cine imaging for volume, function, mass, and wall thickness. Long-axis and axial cine stacks are specifically useful to assess for regional wall motion abnormalities. Post-contrast imaging also plays a role in RV assessment, though its value is often limited due to a thin RV wall, which often prohibits a reliable accumulation of contrast. Flow imaging with modified stacks is important to identify and quantify shunts such as atrial (ASD) and ventricular (VSD) ones (Figure 12) [138].

Cardiac MRI is also useful for LV assessment in CHD, similarly to the RV. It plays a key role in evaluating LV volume, function, and mass. LGE imaging plays a more important role in the LV. Non-ischaemic fibrosis is at times observed in surgically corrected CHD [114,137]. This is especially relevant when athletes present with LV dysfunction and/or arrhythmias. The presence of scarring helps in the risk stratification process and allows for more personalised exercise advice and the outlining of appropriate treatment algorithms (medical therapy, ablation, devices).

The threshold for utilising cardiac MRI protocols that screen for adult CHD should be lower in younger athletes when compared to older athletes. Moreover, RV dilatation on echocardiographic screening should prompt a cardiac MRI evaluation of possible shunts. Several adult CHD conditions are considered incompatible with competitive sports, including Eisenmenger syndrome, Ebstein anomaly, secondary pulmonary hypertension, univentricular hearts, congenitally corrected transposition of the great arteries, and surgically corrected transposition of the great arteries (corrected by the Mustard, Senning, or Rastelli procedure). Cardiac MRI plays a key role in personalised exercise recommendations in athletic individuals harbouring less complex adult CHD phenotypes. This may include the assessment of RV function and volumes in patients with an ASD or RV outflow tract obstruction, LV function and volume in patients with a VSD or patent ductus arteriosus (PDA), and aortic dimensions, and the evaluation of residual coarctation following repair and to rule out fibrosis in repaired Tetralogy of Fallot [135,137,139].

Among athletes, cardiac MRI-derived RV volume reference ranges should be adjusted for physiological adaptation to exercise. Although evidence remains heterogeneous and scarce, some studies have attempted to bridge the gap and provide RV function and volume reference ranges for athletes [30,31,32,113].

## 16. Ischaemic Heart Disease

Ischaemic heart disease (IHD) is more prevalent among middle-aged and older athletes, and its diagnosis should be prompted in individuals presenting with symptoms of myocardial ischaemia. Despite regular exercise reducing the overall risk of acute coronary syndromes, studies have reported a higher prevalence of atherosclerotic plaques in athletes compared to sedentary males [140,141].

Cardiac MRI has two potential roles among athletes suspected of having IHD: direct coronary imaging, and the detection of myocardial ischemia in response to pharmacologic stress agents [142,143,144]. The pharmacological agents commonly used include adenosine, regadenoson, or dobutamine. The latter is also able to detect wall motion abnormalities following inotropic stress. Adenosine and regadenoson detects perfusion defects in response to vasodilator stress and are favoured in cases of arrhythmias such as ventricular tachycardia [145]. Regadenoson is delivered via a single bolus injection, compared to the infusion required with adenosine. It is preferred over adenosine in cases of asthma or chronic obstructive pulmonary disease, as it is not associated with severe bronchospasm [146].

Computed tomography (CT) remains the gold standard for non-invasive coronary imaging; however, cardiac MRI provides a radiation-free alternative if ischaemic testing/myocardial viability is the clinical question. Apart from this, a cardiac MRI’s quality is not affected by coronary calcification. Compared to the iodinated CT contrast agent, gadolinium-based contrast agents are not nephrotoxic; however, their use is discouraged in cases of acute kidney injury or severe chronic kidney disease (an estimated glomerular filtration rate [eGFR] < 30 mL/min/1.73 m^2^) due to the low risk of nephrogenic systemic fibrosis [147]. The diagnostic accuracy of three-dimensional navigator-gated MRI has been found to be comparable to that of CT, with MRI demonstrating a sensitivity of 75% and specificity of 77%, versus 82% and 79% for CT, respectively (both differences were found to be statistically non-significant). This makes MRI an increasingly viable imaging modality [148,149].

While coronary imaging may assess the presence of CAD, the primary determinant of SCD risk in affected athletes is myocardial ischaemia. Therefore, functional imaging provides crucial prognostic information [150]. Various modalities are available, with stress echocardiography being one of the preferred choices among athletes, as it is radiation-free and does not require any administration of pharmacological agents if an exercise protocol is used [9]. Nonetheless, adenosine, regadenoson, and dobutamine perfusion cardiac MRI are also viable, radiation-free options, particularly in athletes with poor echocardiographic windows. A recent meta-analysis has also shown a superior test accuracy for perfusion CMR when compared to stress echo (dobutamine) (sensitivity 0.88 vs. 0.72, specificity 0.84 vs. 0.89) [151]. Cardiac MRI, however, also provides additional information, including the assessment of myocardial scarring and viability [152].

**Figure 13:**
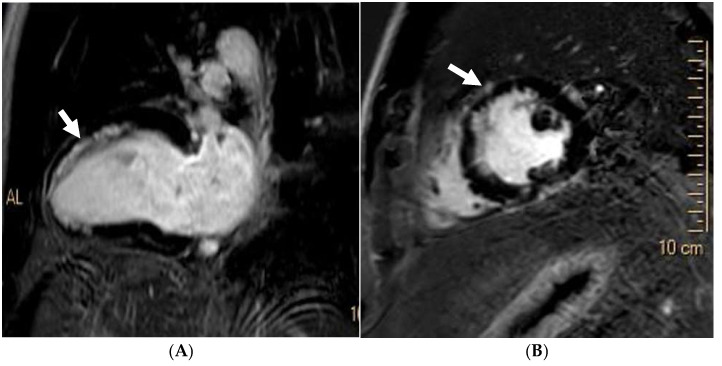
A 52 year old North African male soccer player who had presented with acute coronary syndrome, requiring PCI to LAD 10 years before. He was referred for risk stratification. A cardiac MRI showed evidence of an LAD infarct (50% thickness) (arrows) (**A**,**B**) with preserved LV ejection fraction.

Stress CMR protocols include b-SSFP in long- and short-axis views, as well as myocardial tagging. During adenosine stress testing, gadolinium contrast is administered in the final minute of infusion to detect perfusion defects. Rest perfusion imaging is only performed if abnormalities are observed during stress perfusion [153,154]. For both dobutamine and adenosine stress, LGE imaging may be acquired to assess for previous infracts and scarring (Figure 13). Real-time imaging has improved the imaging quality when breath holding is an issue. Athletes with bradycardia may have issues with breath holding for long periods of time, and this technique may therefore be useful [152]. Athletes with CAD may be permitted to compete on a case-to-case basis, provided they remain symptom-free and exhibit no inducible ischaemia [9,10].

## 17. Myocardial Infarction with Non-Obstructive Coronaries

CMR plays a pivotal role in the evaluation of myocardial infarction with non-obstructive coronaries (MINOCA), often revising an individual’s diagnosis [155,156]. The most common underlying diagnoses in MINOCA include myocarditis, true myocardial infraction that may be secondary to embolic events or spasm, and takotsubo cardiomyopathy [156,157]. MINOCA may also be representative of a true myocardial infarct in the presence of recanalised non-obstructive plaque. Coronary microvascular dysfunction (CMD) is another potential diagnosis in MINOCA, which can have long-term consequences if not adequately diagnosed and managed [158].

**Figure 14:**
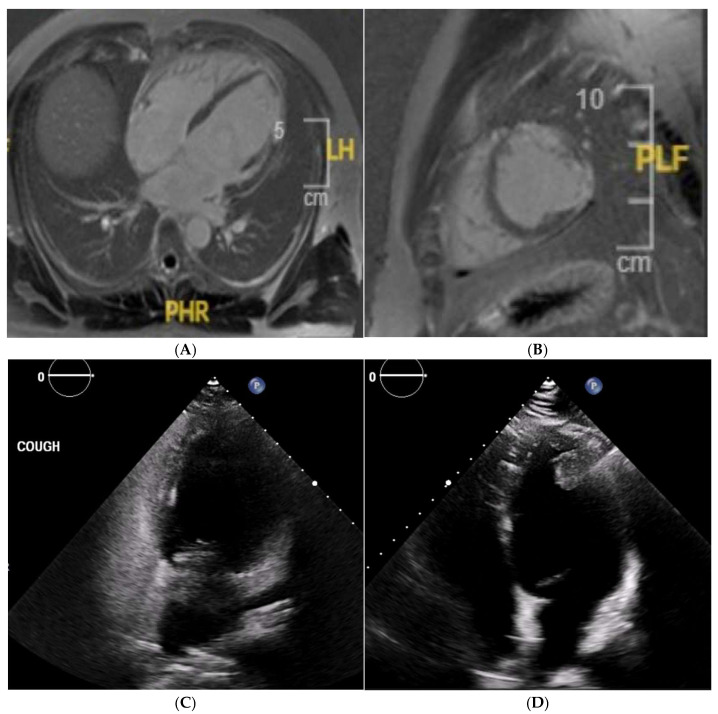
A 21 year old Caucasian male who played soccer professionally at club level. He presented with Troponin-positive chest pain. Invasive coronary angiography was normal. A cardiac MRI showed a mildly reduced LV ejection fraction, together with a transmural LAD infarct involving the anterior and lateral wall (**A**,**B**). A bubble echocardiographic study confirmed the presence of a large patent foramen ovale (**C**). This was corrected percutaneously, though later again presenting with another MINOCA presentation, complicated with a large LV thrombus (**D**).

Different cardiac MRI sequences are key components in a MINOCA evaluation. Cine imaging offers a morpho-functional assessment, specifically evaluating global and regional systolic function, as well as chamber dimensions. T1 mapping provides tissue characterisation and is essential in assessing myocardial injury. T2 mapping and T2-weighted sequences are useful in detecting myocardial oedema. Post-contrast imaging (LGE) is performed to identify extracellular volume expansion or replacement fibrosis (Figure 14). These techniques complement each other, providing a comprehensive evaluation of myocardial injury. CMD may also be assessed using quantitative stress CMR perfusion mapping, which may identify CMD as a reduced global stress perfusion and/or myocardial perfusion reserve (MPR). The use of CMR for suspected CMD is particularly valuable in centres lacking the expertise or resources for physiological testing during invasive coronary angiography [159,160].

MINOCA carries a better prognosis than myocardial infarction with obstructive coronary artery disease at invasive angiography, which is an important consideration when evaluating an athlete’s return to sports [161]. Furthermore, a normal CMR result is associated with a better prognosis [162]. While evidence for returning to sports following MINOCA remains limited, guidelines recommend a similar strategy to chronic coronary syndrome (CCS), which allows athletes to resume sports if they remain asymptomatic, demonstrate normal electrocardiogram and echocardiographic findings, and show no signs of ischaemia on functional imaging [9,10]. Inflammatory disorders presenting as MINOCAs are discussed in a subsequent section.

## 18. Coronary Artery Anomalies

Coronary artery anomalies (CAAs) are congenital abnormalities affecting the origin or course of one of the three main coronary arteries [163]. This broad category encompasses conditions including anomalous aortic origin of a coronary artery (AAOCA), anomalous coronary artery from the pulmonary artery (ACAPA), coronary fistulae, and myocardial bridging [135,164]. The prevalence of CAA ranges from 0.21% to 5.97% among the general population and is on the rise due to increased availability and advancements in cardiac imaging resulting in higher detection rates [11,165,166].

The diagnosis of CAA is crucial among athletes as it has been associated with SCD [11,155,156,167,168]. Among CAAs, an anomalous left coronary artery origin has been associated with the highest risk [169]. Other anatomical types of CAA also confer a risk of SCD. These include an acute-angled take-off from the aorta, proximal narrowing of the anomalous vessel, a slit-like ostium, anomalous coursing between the aorta and pulmonary artery (inter-arterial course), and an intramural course [169,170]. The detection of CAA is particularly relevant in younger athletes, similar to other congenital heart disease [171]. Therefore, a cardiologist should maintain a high index of suspicion among younger athletes presenting with symptoms of ischaemia, such as exertional chest pain or syncope [172].

Echocardiography may suffice to establish the origin of coronary arteries; however, more anatomic confirmation may be required to further delineate CAA. This mainly includes both CT and cardiac MRI, keeping in mind that cardiac MRI offers a radiation-free scan, which is more suitable for younger athletes [173,174]. Current European guidelines recommend CT as the first-line imaging modality, while the American Heart Association recommends cardiac MRI as an alternative [135,175].

Cardiac MRI sequences for CAA should focus on navigated cine imaging, with or without a 3D whole-heart coronary MRA. A short-axis cine stack of the aortic root may aid in directly identifying the coronary origin [174]. Bradycardia in athletes can pose challenges, resulting in a long acquisition time and issues with breath holding. As mentioned, real-time imaging may be a potential solution for this. Among cases of an anomalous circumflex artery, the ‘crossed aorta’ or ‘RAC sign’ on the four-chamber cine view may be used, similar to TTE techniques [176,177]. Perfusion MRI is useful to detect CAA-related ischaemia, which may alter the management [178,179]. Furthermore, LGE imaging may reveal the presence of fibrosis, which has been linked with CAA or scarring [180,181].

Eligibility for competitive sports depends on symptoms, LV function, the presence of ischaemia, fibrosis, and the specific anatomical variant of CAA [9]. Patients with high-risk features should be restricted from competitive sports unless surgical correction is performed, particularly if symptomatic [9,135,182].

## 19. Myocarditis

Myocarditis has a prevalence of approximately 10.2 to 105.6 per 100,000 worldwide [183]. Before the COVID-19 pandemic, the highest risk was observed in adults aged 20–40 years, more commonly in males [184]. Although endomyocardial biopsy remains the gold-standard diagnostic method, there has been a growing clinical shift toward non-invasive cardiac MRI, which provides valuable diagnostic insights [185,186]. The Lake Louise criteria have provided diagnostic cardiac MRI criteria for myocarditis, which have become an integral part of cardiac MRIs’ interpretation [185].

**Figure 15:**
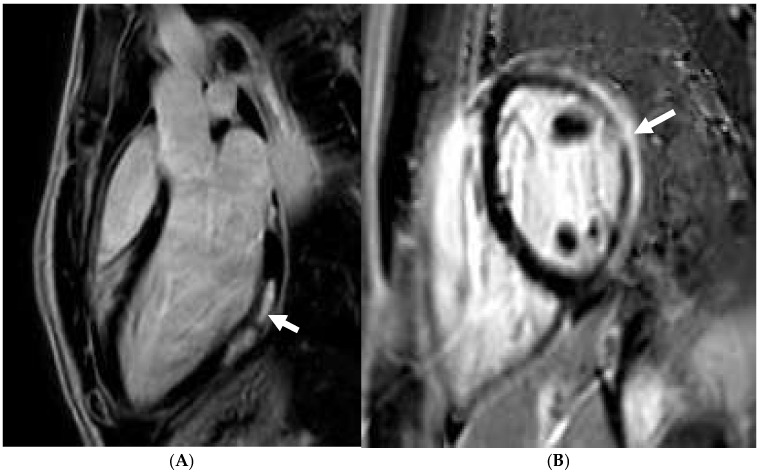
A 17 year old Caucasian male who played handball at club level presented with recurrent myocarditis. His father, a former endurance athlete, presented with an out of hospital cardiac arrest, was implanted with a defibrillator, and was genetically positive for a likely pathogenic DSG2 variant. Cardiac MRI in the patient in question showed evidence of an extensive subepicardial high signal extending into the anterior and lateral wall (arrows), suggestive of persistent replacement fibrosis (**A**,**B**). He unfortunately refused to undergo genetic testing.

Recent evidence also suggests that arrhythmogenic cardiomyopathy may have a similar clinical presentation. The new hot phase/remission concept in arrhythmogenic cardiomyopathy has generated a lot of academic interest and is now an important differential diagnosis in the most recent updated guidelines [187]. One should certainly entertain the possibility of a hot phase in cases of persistent ventricular arrhythmias, a family history of SCD, or chronically elevated Troponin levels. This ‘hot phase’ presentation may be the first clinical manifestation of NDLVC (Figure 15) [188]. Cardiac MRI is one of the multi-modal investigations that is often required in such cases, together with ECG, echocardiography, and endomyocardial biopsy. Patterns of LGE may prompt such diagnoses, and this includes the non-ischaemic LGE distribution in subepicardial inferolateral and septal regions, as well as a circumferential subepicardial ‘ring-like’ pattern, which is typical in DSP, PKP2, and FLNC gene mutations [189,190,191].

**Figure 16:**
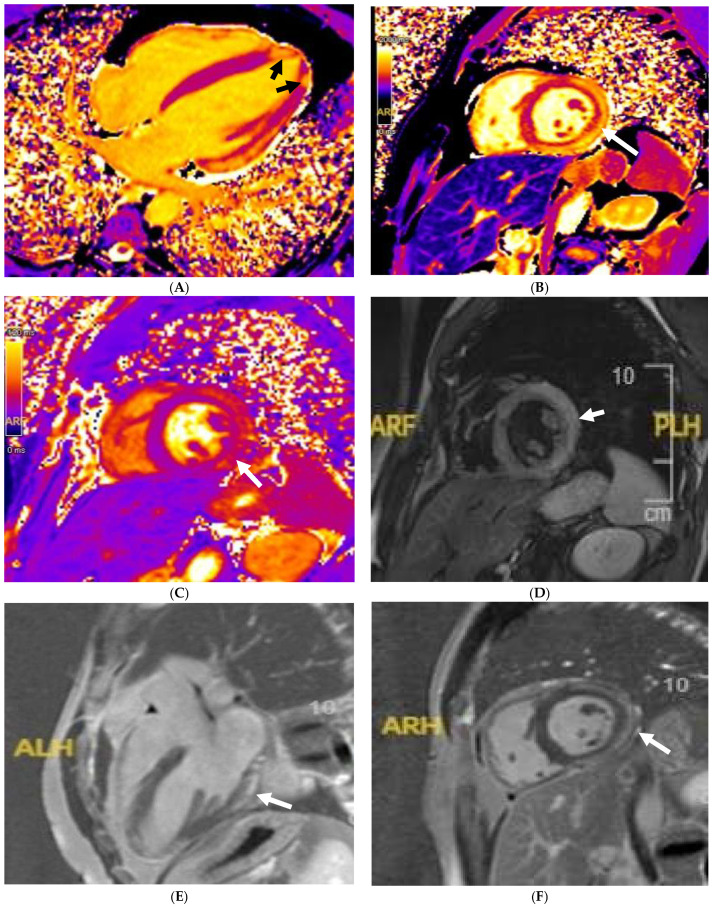
A 15 year old Caucasian male who played basketball at club level, presenting with chest pain, fever, and Troponin elevation. Cardiac MRI showed evidence of myocardial injury (**A**,**B**) on T1 mapping, with myocardial oedema on T2 mapping (**C**) and STIR (**D**) imaging (arrows). Post-contrast imaging showed evidence of extracellular volume expansion, with subepicardial late-gadolinium enhancement in the anterior and lateral wall (**E**,**F**) (arrows). The findings satisfied Lake Louise Criteria for myocarditis.

The cardiac MRI sequences used for myocarditis diagnosis include b-SSFP for ventricular function, T2-weighted triple inversion recovery imaging for oedema, T1-weighted fast spin echo imaging for hyperaemia and myocardial injury, and LGE imaging for scar evaluation (Figure 16) [192,193]. Oedema, fibrosis, and necrosis result in increased extracellular space during the acute phase of myocarditis.

Cardiac MRI plays a vital role in the follow-up assessment of athletes, particularly to evaluate myocardial scarring and determine the safety of athletes returning to sports. Expert consensus statements previously recommended a repeat cardiac MRI within 3–6 months to re-evaluate for ongoing myocardial oedema and injury. However, recent data from SARS-CoV-2 infection suggest that recovery can occur as early as 4–6 weeks from presentation [194]. The most updated recommendations now support an earlier re-evaluation with CMR and a possible return to sports in the absence of high-risk clinical and imaging features [9,195].

The persistence of replacement fibrosis in the absence of ongoing myocardial oedema should prompt discussion with an expert. Eligibility for return to competitive sports should be adjudicated on a case-by-case basis, complemented with an in-depth evaluation that consists of symptoms, cardiac biomarkers, LV systolic function, and reproducible arrhythmia [9]. Among athletes with pericarditis, patients must demonstrate complete clinical recovery, with no evidence of ongoing pericardial inflammation, before resuming competitive sports. Return is generally permitted after 30 to 90 days of symptoms’ resolution [9,195].

## 20. Pericarditis

The incidence of pericarditis is on the rise, with a recent study showing a notable rise from 7.2 to 29.2 cases per 100,000 between 2000 and 2019 [196]. Similar to myocarditis, pericarditis also displays characteristic appearances on cardiac MRI, typically with signs of pericardial oedema and thickening, with or without a constrictive physiology. CMR imaging is considered a supportive tool in the diagnosis of pericarditis [197].

CMR protocols for pericarditis are similar to myocarditis, and physicians may rely on CMR to exclude a corresponding myocarditis. The sequences include b-SSFP cine imaging for the assessment of ventricular function and pericardial effusion, T1-weighted imaging to assess for pericardial thickening, T2-weighted imaging for pericardial oedema, and LGE imaging for pericardial fibrosis.

Cardiac MRI may play an important role in the follow-up assessment of athletes suffering from pericarditis, particularly when chronic pericardial inflammation or constriction is suspected [198]. Patients must demonstrate a complete clinical recovery, with no evidence of ongoing pericardial inflammation (based on symptoms, inflammatory markers, and the exclusion of pericardial effusion), before resuming competitive sports. Return is generally permitted after 30 to 90 days of symptoms’ resolution [9,197].

## 21. Conclusions

Cardiac MRI plays a key role in the evaluation of athletes. It helps to offer a more personalised management strategy, allowing athletes to be better informed of the risks in a shared decision manner. Cardiac MRI plays a key role at discriminating between physiology and pathology. This imaging modality has also evolved, with several new sequences and groundbreaking techniques now available for a more comprehensive cardiac assessment. Standardisation across institutions, and an adequate representation of all athlete subgroups, irrespective of discipline, gender, and race, is still lacking. Our understanding of the role and impact of cardiac MRI will undoubtedly evolve over time. Spearheading longitudinal studies will help address the paucity of data and will inevitably improve our understanding of the physiology and disease in athletes.

## Data Availability

Not applicable.
